# Synchronous solid neuroendocrine breast carcinoma and abdominal lymphoma: A case report and review of the literature

**DOI:** 10.3892/ol.2012.1044

**Published:** 2012-11-23

**Authors:** MIGUEL ALONSO-RUANO, EUGENI LÓPEZ-BONET, MARIA VICTORIA HUERTA-ANAYA, ESTER VILA-CAMPS, LUIS BERNADÓ, FRANCESC TUCA-RODRÍGUEZ, PEDRO SUAREZ-PUMARIEGA, JAVIER A. MENENDEZ

**Affiliations:** 1 Departments of Gynecology, Dr Josep Trueta Hospital of Girona, Girona; Catalonia, Spain; 2 Anatomical Pathology, Dr Josep Trueta Hospital of Girona, Girona; Catalonia, Spain; 3 Department of Anatomical Pathology, Figueres Hospital, Figueres; Catalonia, Spain; 4 Department of Gynecology, Santa Caterina Hospital; Catalonia, Spain; 5 Catalan Institute of Oncology (ICO); Catalonia, Spain; 6 Girona Biomedical Research Institute (IDIBGi), Catalonia, Spain

**Keywords:** neuroendocrine tumors, neuroendocrine breast carcinomas, second primary malignancies, lymphoma

## Abstract

Neuroendocrine tumors (NETs) are frequently associated with second primary malignancies (SPMs). Earlier studies have demonstrated that NETs are highly associated with synchronous or metachronous gastrointestinal and genitourinary SPMs. We report, for the first time, a case of pure NE breast carcinoma (NEBC) exhibiting all of the World Health Organization (WHO)-categorized morphological and phenotypic NE features (i.e., round solid nests of spindle cells, plasmacytoid cells, large clear or mucinous signet-ring cells with a peripheral palisading tendency and immunohistochemical positivity for the NE markers synaptophysin and chromogranin in more than 50% of the tumor cell population) along with synchronous abdominal non-Hodgkin’s lymphoma. In the present study, we review the diagnosis, clinicopathological features and histogenetic profiling of NEBC and discuss the literature relevant to the clinical and anatomopathological management of this case. This previously unreported case of synchronous solid NEBC and abdominal lymphoma, together with earlier studies showing that primary symptoms are caused by SPMs in a significant subgroup of NET patients, strongly supports the notion that NETs should be cautiously considered to be index tumors. Therefore, risk-adapted clinicopathological follow-up with systematic investigation is strongly recommended.

## Introduction

The occurrence of synchronous or metachronous second primary malignancies (SPMs) is increased in patients with neuroendocrine tumors (NETs) compared to the general population (1,2). Since primary NETs of the breast are extremely rare (3–8), evidence is lacking as to whether patients with NE breast carcinomas (NEBC) could also suffer the development of a second primary malignancy. In the present study, for the first time, we report a case of pure NEBC accompanied with synchronous abdominal non-Hodgkin’s lymphoma. We review the diagnosis, clinicopathological features and histogenetic profiling of NEBC and discuss the literature relevant to the clinical and anatomopathological management of this case. Written informed consent was obtained from the patient for publication of this case report and accompanying images.

## Case report

**Figure 1.**
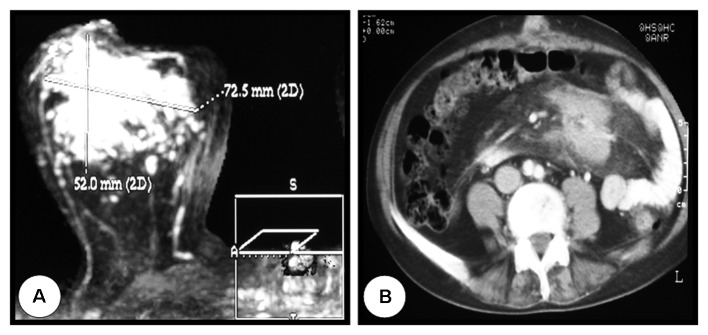
Synchronous solid neuroendocrine breast carcinoma and abdominal lymphoma. (A) Magnetic resonance image (MRI) showing a 7-cm mass that is highly suggestive of neuroendocrine breast carcinoma (NEBC) in the upper outer quadrant of the patient’s right breast; (B) Computed tomography (CT) scan of the abdomen showing a mesenteric mass of 6 cm (i.e., synchronous abdominal non-Hodgkin’s lymphoma).

**Figure 2.**
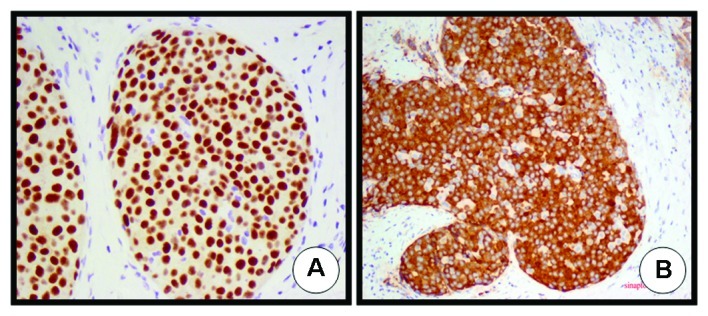
Immunohistochemical features of neuroendocrine breast carcinoma (NEBC). Immunohistochemistry of NEBC showing positivity for (A) estrogen receptor and (B) synaptophysin.

A 58-year-old woman presented with a lump in her right breast. A physical examination revealed an extensive and irregular mass located in the upper outer quadrant of the patient’s right breast. Mammography and magnetic resonance imaging (MRI) showed a 7-cm mass that was highly suggestive of malignancy (Fig. 1A), and pathology revealed positive axillary lymph nodes. Core needle biopsy identified the mass as an estrogen receptor (ER)-positive (ER+) infiltrating ductal carcinoma. Further investigation using computed tomography (CT) revealed an abdominal mesenteric mass of 6 cm, which was biopsied and diagnosed 1.5 months later as a nodular, low-grade (grade I) follicular lymphoma (Fig. 1B). To avoid undesirable treatment of the mesenteric mass with conventional chemotherapy and since the patient had undergone mitral valve replacement 7 years earlier, she was treated with letrozole (2.5 mg/day) in a neoadjuvant setting for four months. Following this therapeutic management, partial clinical response was observed in the breast tumor with no change in the mesenteric tumor. Four months later, the patient underwent total mastectomy with axillary lymph node dissection. Histological examination revealed two solid grade II neuroendocrine tumors (NETs) that measured 5.5 and 0.9 cm, accompanied by severe lymphovascular invasion and 14 positive metastatic lymph nodes out of 26. A histological assessment revealed the following immunohistochemical features: ER 3+ (Fig. 2A), progesterone receptor (PR) 1+, HER2- 1+, p53 negative, E-cadherin 3+ and Ki67 3+. Staining was positive for both specific markers of NETs (i.e., chromogranin and synaptophysin; Fig. 2B),

## Discussion

NE features have been recognized in human breast tumors for many years. Breast cancer-associated NE features may be detected either as scattered cells immunoreactive for NE markers in carcinomas of the usual type or as a special type of tumor in which the vast majority of the cells display NE characteristics (3,4). In 1977, the first eight cases of breast tumors classified as NETs based on the presence of argyrophilia and cytoplasmic dense core granules were published (5). In 1989, Pagotti et al (6) reported that approximately 8% of breast tumors displayed some degree of NE differentiation in a consecutive series of 100 infiltrating breast carcinomas. However, the actual prevalence of primary pure NE-differentiated breast carcinoma (NEBC) was less than 1%. A retrospective review of the mammograms of 1,845 histopathologically proven breast cancer cases revealed five NEBCs (0.3%) (4). In 2003, the WHO classification of breast tumors established that NEBC should exhibit morphological features similar to those of NE tumors of the gastrointestinal tract and lungs together with the immunohistochemical expression of NE markers (i.e., chromogranin and synaptophysin) in more than 50% of the tumor cell population (7). The latter is a unique requirement for the accurate diagnosis of primary pure NEBC. When utilizing the previous WHO classification to determine the prevalence of NEBC in our institution (Dr Josep Trueta University Hospital, Girona, Spain), we found that only 7 out of 1,368 infiltrating breast tumors fully satisfied the NEBC criteria (0.5%) (8). This level of NEBC incidence does not significantly differ from that reported in earlier studies.

Although the prevalence of pure NEBC remains to be definitively established when strictly following the WHO criteria, there is an urgent need to establish NEBC-associated clinico-histopathological features, prognostic factors and/or imaging patterns that are distinct from those of other BC subtypes. It has been reported that the presentation of NEBC is accompanied by fairly well-circumscribed dense round or irregular masses with spiculated or lobulated margins and homogeneous enhancement with a time-intensity curve on MRI (3,4,9). The NE histological features of pure NEBC are similar to those observed in NE tumors at other body locations. One of the primary features of NEBC is related to the presence of tumor cells in round solid nests of spindle cells, plasmacytoid cells or large clear or mucinous signet-ring cells with a peripheral palisading tendency. Rarely, NEBCs exhibit polarized arrangements of tumor cells containing eosinophilic granules around the lumina. Together, these histological features form rosette-like structures in a carcinoid-like pattern along with a cordonal arrangement of the infiltrating tumor cells. However, the solid nests may also be found in the solid type of in situ or infiltrating lobular carcinoma, making an accurate diagnosis of NEBC more difficult. In our case, this alternative diagnosis was excluded due to the presence of rounded cells arranged in solid nests both in the first biopsy and in the surgical specimen when assessing the palisading cells for E-cadherin positivity and p63 negativity. Therefore, when the diagnosis is suggestive of NEBC, the ultimate diagnosis should be based on the immunohistochemical expression of one or both of the NE markers synaptophysin and chromogranin in more than 50% of the BC cell population (10). Both markers were found in our case. It should be noted that we observed a diffuse but strong staining for synaptophysin, whereas the chromogranin staining was weak with a focal distribution. The weak chromogranin staining may be related to the fact that most diagnostic laboratories provide monoclonal antibodies raised against isoform A of chromogranin. Accordingly, if an NEBC primarily expresses isoform B of chromogranin, the tumor will be scored as chromogranin-negative when using an antibody that exclusively recognizes isoform A.

NETs are frequently associated with synchronous or metachronous second primary malignancies (SPMs). It has been reported that almost 15% of patients with NETs can be identified as having an SPM (1,2). Prommegger et al (1) reviewed fourteen patients with NETs and synchronous or metachronous SPMs from a series of 96 patients with NETs to determine the primary site and characteristics of the NETs and associated SPMs. Regardless of the localization of the NET (i.e., appendix, ileum, duodenum, stomach, jejunum, pancreatic tail, rectum or lung), the authors found that three months to five years after diagnosis, NETs were highly associated with gastrointestinal and genitourinary SPMs (i.e., SPMs of the colon, stomach, bladder, ovary, pancreas, breast, lung, gastric MALT lymphoma and liver). However, Prommegger et al (1) did not report any patients with NEBCs. In our case, although we considered the possibility of metastatic BC when analyzing the mesenteric biopsy, the immunohistochemical profile clearly revealed a nodular, low-grade (grade I) follicular lymphoma (CD20+, CD10+, BCL2+, CD23+, CD3−, CD5− and cyclin D1−). It should be noted that although the occurrence of second tumors is a well-recognized phenomenon in BC patients who have undergone adjuvant chemotherapy and radiotherapy, presentation with second synchronous non-breast primary malignancy is extremely rare. Tanaka et al (11) reported a significantly increased risk (30%) of the development of ovarian cancer, thyroid cancer and non-Hodgkin’s lymphoma among BC patients relative to the risk of the general population. However, this sequence of events typically involves an interval of several years. The synchronous presentation of BC and an SPM, that is, a malignancy diagnosed within a six-month period, is an exceptional phenomenon, particularly when considering the synchronous association of BC and lymphoma. Although there are a few publications describing this specific association, all of these publications report that the lymphoma was either located in the breast itself or in the axillary nodes (11–13). The exceptionality of our case is that the lymphoma occurred in the abdominal cavity and, in addition, that the lymphoma was synchronous with a very rare subtype of BC (i.e., NEBC). These findings strongly support the notion that we should confirm or reject a differential diagnosis of a SPM. Awareness of this may greatly improve the staging and treatment of both diseases - which may be different - when there is a diagnostic suspicion of metastatic disease in patients with NEBC.

Prognostic factors in NEBC do not differ from those classically considered for other BC subtypes. Histological grade, mucinous differentiation and the expression of ER and PR have been suggested as reliable features that are indicative of the clinical outcome of NEBC (14,15). In agreement with earlier studies, the tumor in our case was classified as grade II (moderately differentiated) according to modified Scarff-Bloom-Richardson histological grading criteria. Although the focal amount of mucinous differentiation was not sufficient for the tumor to be considered a mucinous carcinoma, it may correlate with a good prognosis, whereas the presence of the small cell NE subtype has been reported to negatively impact the prognosis of NE tumors. The NEBC case described herein was positive for both ER and PR. Regarding treatment, anthracycline-based chemotherapy is the first choice, and maintenance hormone therapy has been generally prescribed for the management of patients with ER/PR-positive NEBC (16). For our patient, we initially used letrozole as hormone therapy in a neoadjuvant setting (2.5 mg/day for four months) due to the tumor size (7 cm) and to avoid the undesirable treatment of the mesenteric mass with conventional chemotherapy. Letrozole was also selected because the patient had been treated with anthracycline-based regimens when she underwent mitral valve replacement 7 years earlier.

We cannot confirm whether there was a direct correlation between the two primary malignancies observed in our patient or whether they were the result of independent events (17). Using array and metaphase comparative genomic hybridization (CGH) with synchronous primary breast tumors, Ghazani et al (18) recently suggested that synchronous BC may represent a special subtype of breast tumor in which, at least in certain cases, one tumor gives rise to the other. Although we are aware that the more widespread clinical use of this technology will require the use of standardized methods for the routine analysis of clinical specimens, CGH arrays should be considered as a valuable tool that may offer a definitive answer during the clinicopathological follow-up of NETs and associated SPMs (19). Since primary symptoms are caused by SPMs in a significant subgroup of NET patients (1), it is reasonable to suggest that NETs and NEBCs should be cautiously considered to be index tumors. Therefore, risk-adapted clinicopathological follow-up with systematic investigation is strongly recommended.
